# Highly Stable 4.6 V LiCoO_2_ Cathodes for Rechargeable Li Batteries by Rubidium‐Based Surface Modifications

**DOI:** 10.1002/advs.202202627

**Published:** 2022-10-17

**Authors:** Tianju Fan, Yujie Wang, Villa Krishna Harika, Amey Nimkar, Kai Wang, Xiaolang Liu, Meng Wang, Leimin Xu, Yuval Elias, Hadar Sclar, Munseok S. Chae, Yonggang Min, Yuhao Lu, Netanel Shpigel, Doron Aurbach

**Affiliations:** ^1^ Department of Chemistry Bar‐Ilan University Ramat‐Gan 5290002 Israel; ^2^ School of Materials and Energy Guangdong University of Technology Guangzhou Guangdong 510006 China; ^3^ Ningde Amperex Technology Limited Ningde Fujian 352100 China

**Keywords:** 4.6V LCO, high voltage batteries, LiCoO2, Li‐ion cathodes, surface coatings

## Abstract

Among extensively studied Li‐ion cathode materials, LiCoO_2_ (LCO) remains dominant for portable electronic applications. Although its theoretical capacity (274 mAh g^−1^) cannot be achieved in Li cells, high capacity (≤240 mAh g^−1^) can be obtained by raising the charging voltage up to 4.6 V. Unfortunately, charging Li‐LCO cells to high potentials induces surface and structural instabilities that result in rapid degradation of cells containing LCO cathodes. Yet, significant stabilization is achieved by surface coatings that promote formation of robust passivation films and prevent parasitic interactions between the electrolyte solutions and the cathodes particles. In the search for effective coatings, the authors propose RbAlF_4_ modified LCO particles. The coated LCO cathodes demonstrate enhanced capacity (>220 mAh g^−1^) and impressive retention of >80/77% after 500/300 cycles at 30/45 °C. A plausible mechanism that leads to the superior stability is proposed. Finally the authors demonstrate that the main reason for the degradation of 4.6 V cells is the instability of the anode side rather than the failure of the coated cathodes.

## Graphical abstract

Enhanced stability of 4.6V LiCoO_2_ electrodes is achieved through application surface coating consisting of Rb, Al, and F. This coating enables effective protection of the cathode's particles from parasitic attacks and promotes the formation of robust passivation layers (SEI). Using fluorinated electrolyte solution, a high capacity of 220 mAh g^−1^ and impressive long‐term stability (500 cycles with 80% capacity retention) are demonstrated.

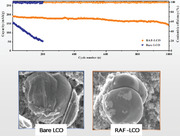

## 1 Introduction

More than three decades after the commercialization of the widely used Li‐ion batteries which are based on LiCoO_2_ (LCO) cathodes, their energy density is still far below the theoretical limit. Traditionally, to avoid structural instabilities and safety risks, the upper cut‐off voltage of LCO cathodes was limited to 4.2 V, which resulted in cells' theoretical (maximal) specific energy density of ≈550 Wh kg^−1^. Over the years, better synthesis methods and incorporation of various dopants and surface coatings significantly improved stability, and currently, LCO cathodes can be charged to 4.4 V providing a maximal theoretical energy density up to ≈670 Wh kg^−1^ Yet, despite this progress, operating Li batteries with LCO cathodes at high voltage is very challenging.

Among the proposed degradation routes, including irreversible phase transformations, Co ions dissolution,^[^ ^1^ ^]^ inhomogeneous Li in/de‐intercalation,^[^ ^2^ ^]^ and surface degradation, the latter appears to be the major obstacle to long‐term stability of high voltage LCO cathodes: upon cycling, the cathodes' surfaces are exposed to parasitic attacks by the electrolyte solutions, leading to dissolution of Co ions^[^ ^3^ ^]^ and loss of surface oxygen.^[^ ^4^ ^]^ The defective sites thus formed facilitate surface cracking^[^ ^5^ ^]^ that exposes fresh zones of the cathode's active mass to further side reactions. Moreover, the high charging potentials induce formation of resistive surface layers (due to irreversible surface phase changes or formation of resistive passivation films), which increase cells' impedance and cause rapid degradation.^[^ ^6^, ^7^ ^]^

In light of this situation, significant efforts were made to stabilize the electrodes–electrolyte solutions interfaces by application of various coating chemistries. As shown in several recent studies, implementation of surface layers such as Li—Al—F,^[^ ^8^, ^9^ ^]^ AlZnO,^[^ ^10^ ^]^ or AlPO_4_@Li_3_PO_4_^[^ ^11^ ^]^ significantly enhanced 4.6 V cathodes stability. Nevertheless, further stabilization of LCO cathodes is required to meet practical demands for the use of high energy density rechargeable Li ion batteries. An effective surface coating should protect the active mass from acidic electrolyte solution species (mostly HF) attacks while allowing fast diffusion/migration of Li‐ions between the cathode's active mass and the electrolyte solution. In this context, an appropriate selection of electrolyte solution and surface‐film‐forming additives is crucial to ensure prolonged high voltage cathodes' operation. As recently shown, improved cycling of 4.6 V LCO was obtained by using fluorinated or sulfonamide‐based electrolytes.^[^ ^12^, ^13^ ^]^ Clearly, an inclusive approach that takes into account both surface and electrolyte solutions modifications, should be adopted.

Here, we report a new surface coating comprising rubidium, aluminum and fluoride ions (RAF), which enables a significantly improved operation of 4.6 V LCO cathodes in Li cells at room and elevated temperatures. The bare and coated cathodes were tested in a conventional electrolyte solution comprising ethyle carbonate/ethyl‐methyle carbonat (EC/EMC 3/7 g g^−1^) in 1 m LiPF_6_ (LP57) and in a (1:1:8 v/v/v) mixture of di‐fluoroethylene/fluoroethylene carbonate/dimethyl carbonate in 1 m LiPF_6_. The use of the latter solution provided excellent stability for various high voltage Li‐ion cathodes and anodes.^[^ ^14^ ^]^

## 2 Results and Discussion

**Figure 1:**
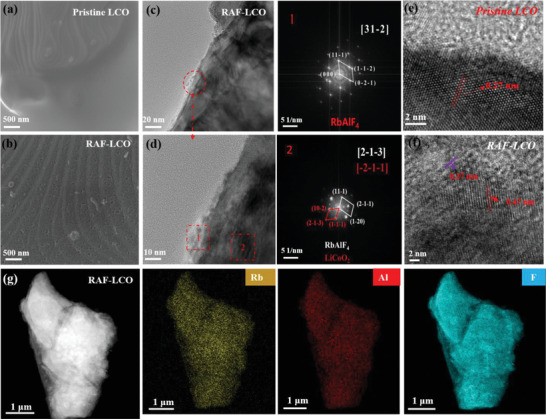
High resolution scanning electron microscopy characterizations of bare and RAF coated LCO particles: SEM images of bare (a) and coated (b) LCO particles. High‐resolution TEM (HR‐TEM) bright‐field images of RAF‐LCO ((c) and (d), 1 and 2 indicate the FFT areas from the RAF‐LCO image d)). HR‐TEM images taken from the surface of bare (e) and coated (f) particles, and g) EDS mapping of a single coated particle.

To obtain a uniform RAF coating with controllable surface composition, commercially available LCO particles were coated using wet‐chemistry routes reported elsewhere (for details see the Experimental Section). The morphological and structural characteristics of the bare and coated LCO particles are shown in **Figure** **1**. As can be seen in the SEM images, in contrast to the smooth surface texture of the bare particles (Figure 1a) the RAF‐coated LCO (Figure 1b) are covered by a thin, porous film. The presence of a layer in an order of tens of nanometers thick on the RAF coated particles is well visualized in the HR‐TEM images shown in Figure 1c,d. Closer inspection of the FFT patterns taken from the surface of RAF coated particles (Figure 1d) reveals the existence of a layered rock‐salt structure on the particle interior and a RbAlF_4_ phase at the external surface. Furthermore, a pronounced increase (by a factor of 1.7) in the surface interplanar spacing was detected for RAF samples. A uniform distribution of all coating elements is well demonstrated by the EDS mapping presented in Figure 1g.

To understand the role of each coating ingredient in the performance of the system, additional coatings comprising RbF and AlF were synthesized. Based on the XRD patterns shown in Figure S1, Supporting Information, one may conclude that the LCO structure remains essentially the same for the bare and coated cathodes. XPS analysis (Figure S2, Supporting Information) displays the presence of Li—F phases in all coated samples, while no changes were observed in the O1s spectra compared to the bare sample. Interestingly, a peak located at ≈73 eV corresponding to AlO*_x_* phases was identified for the AlF coating. In contrast, a peak at higher binding energy attributed to the presence of AlF*_x_* compounds was detected for the RbAlF_4_ coated sample.

**Figure 2:**
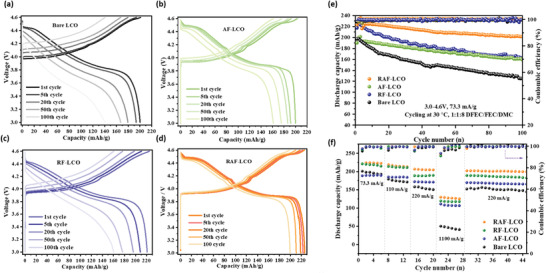
Electrochemical performance of bare and coated LCO cathodes: GCD voltage profiles recorded upon 100 cycles at 73.3 mA g^−1^ (corresponds to 0.3C rate, C = 220 mAh g^−1^) for a) bare and b) AlF, c) RbF, and d) RbAlF coated LCO particles. The long‐term stability measured over 100 cycles at 0.3C rate and the rate capability performance of the examined samples are presented in panels (e) and (f), respectively.

Galvanostatic charge–discharge (GCD) profiles of bare and coated electrodes in DFEC based electrolyte solutions (measured at 73 mA g^−1^) are shown in **Figure** **2**a–d. The corresponding cycling performance and rate capability of all examined systems are presented in Figure 2e,f. As shown in Figure S3, Supporting Information, cycling of bare LCO and RAF coated particles in the standard LP57 electrolyte solution (1.0 m LiPF_6_ in 3:7 EC/EMC ) leads to a rapid capacity fading for both cathodes. Based on previous studies, this behavior can be ascribed to significantly better passivation of both the LCO cathodes and Li anodes (as is later explained) due to the formation of fluorine‐rich surface species such as LiF and Li*_x_* PO*_y_* F*_z_*, which were found to be important building blocks in protecting surface films formed on both cathodes and anodes in Li ion batteries.^[^ ^9^, ^15^ ^]^

The superior stability of the RAF coated LCO over the bare LCO and LCO electrodes with other coatings is well reflected in Figure 2; an initial capacity of 224 mAh g^−1^ and ≈90% capacity retention were obtained after 100 cycles. For the RbF coated LCO electrodes, a similar initial capacity as that of the RAF coated cathodes was obtained, however, ≈28% of capacity was lost after 100 cycles. In contrast, both bare and AlF coated cathodes displayed a significantly lower initial capacity of 200 mAh g^−1^, while the latter showed much better cycling stability with less than 20% loss. The rate capability performance presented in Figure 2f shows that the best performance was obtained for the RAF coated cathodes with a capacity of 125 and 224 mAh g^−1^ at current densities of 73.3 and 1100 mA g^−1^, respectively.

**Figure 3:**
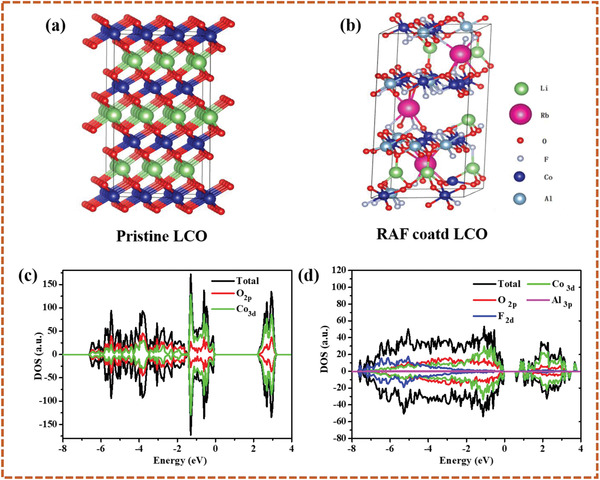
DFT analysis of bare and coated LCO cathodes: Atomic structures of pristine LCO (a) and RAF coated LCO (b) and calculated density of stats spectra for the bare LCO (c) and coated LCO (d).

Based on these observations, three assumptions can be made: 1) clearly, all examined surface coatings increase the LCO cathodes stability; 2) the presence of rubidium yields high capacity values and improves rate capability; and 3) the presence of aluminum is important for long‐term stability (RAF and AlF coated cathodes showed the best capacity retention). While the latter assumption is fully supported by various studies which ascribe this behavior to the formation of a protective layer that mitigates strong side reactions between the electrolyte solutions and the active materials,^[^ ^16^, ^17^, ^18^ ^]^ the role of the Rb in the enhanced capacity has to be further clarified. To address this issue, the structure of the interface between the LCO and the Rb coating was analyzed by density functional theory calculations using the Vienna Ab initio Simulation Package (VASP) (more details related to the theoretical calculations appears in the Supporting Information) assuming a stoichiometry of a Li_2/9_Rb_1/9_Co_2/3_Al_1/3_O_4/3_F_2/3_. This phase has been shown in **Figure** **3**, has a solid solution structure in which the Rb cations are located between the crystalline layers. This results in a significant increase of the C‐lattice from 13.87 Å calculated for the bare LCO to 17.41 Å in the RAF‐coated particles. Note that these changes are only related to the surface of the cathodes, however not the particles’ bulk (as indicated by the presented XRD). These observations suggest that Rb acts as a pillar providing enlarged channels for a facile ionic transport and hence better interfacial kinetics^[^ ^19^, ^20^ ^]^ The calculated density of states (DOS) of both pristine and coated LCO are shown in Figures 3c and 3d, respectively (individual spectra are presented in Figure S4, Supporting Information). The bandgap of pristine LCO was found to be 2.22 eV, in good agreement with recently reported values.^[^ ^21^ ^]^ As seen, compared to the density of states (DOS) spectrum of the bare LCO less sharp spectrum was obtained for the RAF coated cathode due to its deformed structure. Nevertheless, a substantially reduced bandgap of 0.88 eV was calculated for the coated LCO implying better electrical conductivity of the solid solution structure associated with faster‐charging kinetics as compared to the unmodified LCO.

**Figure 4:**
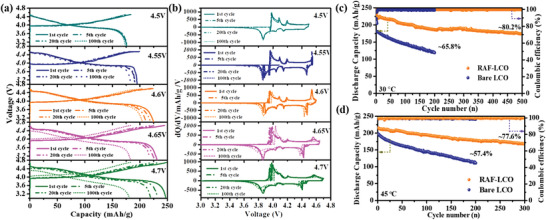
The influence of the upper charging cut‐off voltage on the performance of RAF‐coated LCO electrodes: GCD and d*q*/d*V* versus *V* profiles obtained over 100 cycles ((a) and (b), respectively). The long‐term performance of the 4.6 V cathodes at 30 °C and 0.5C and at 45 °C and 0.3C are presented in panels (c) and (d), respectively.

Encouraged by the superior behavior of the RAF‐coated cathodes, further electrochemical measurements were performed. The GCD voltage profiles and the differential capacities of the coated electrodes were recorded throughout 100 cycles at 0.5C rate and are presented in **Figures** **4**a and 4b, respectively. A comparison of the cycling performance as a function of maximal potentials is presented in Figure S5, Supporting Information. As can be seen, charging to 4.5 V results in a discharge capacity of 176 mAh g^−1^ and excellent capacity retention of 98.6% after 100 cycles. Raising the charging voltage to 4.55 V increases the capacity by more than 10%, albeit with slightly lower stability (94% after 100 cycles), while a higher maximal charging voltage of 4.6, 4.65 and 4.7 V results in a higher discharge capacity of 224, 231 and 247 mAh g^−1^, respectively. This, however, comes at the expense of stability, in particular for the cathodes charged to 4.65 and 4.7 V, which showed poor capacity retention after 100 cycles of 82% and 76%, respectively. Yet, considering the chemical and structural instabilities associated with such high voltage,^[^ ^8^, ^22^ ^]^ these values are far from trivial and demonstrate the protective nature of the RAF coating even at high charging potentials, which usually lead to much faster degradation.^[^ ^23^, ^24^ ^]^

Focusing on the cycling performance at 4.6 V, the long‐term performance of the coated and bare electrodes at 0.5C (see Figure 3c) shows a fast capacity fading for the non‐coated cathode, with less than 70% of the initial capacity retained after 200 cycles. In contrast, the RAF‐coated electrode exhibits excellent cyclability with more than 80% capacity retention after 500 cycles. The stability of the RAF‐coated cathodes was further evaluated at an elevated temperature of 45 °C. As shown in Figure 3d, an impressive cyclability was achieved throughout 300 cycles with more than 77% capacity retention, while rapid degradation expressed by more than 40% capacity loss was observed for the bare LCO electrodes. As can be seen in Table S1, Supporting Information, these results present significantly improved performance compared to the recent studies related to 4.6 V LCO cathodes.

**Figure 5:**
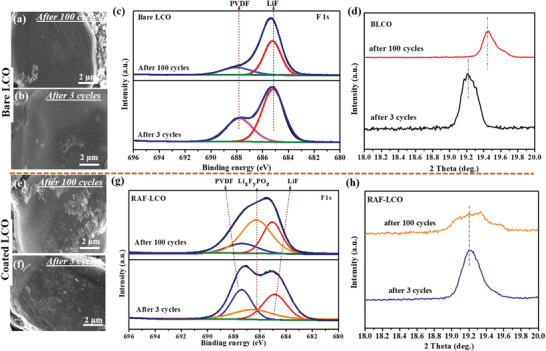
Post‐mortem analysis of bare and coated LCO cathodes after 3 and 100 cycles: SEM images (a,b,e,f) and XPS and XRD data of bare (c,d) and Rb—Al—F coated (h,i) samples.

Further insights into the stabilization mechanism of the high voltage LCO cathodes enabled by the Rb—Al—F coatings were obtained by post‐mortem analyses including XPS, XRD, and SEM. As shown in **Figure** **5**, all measurements were performed at the end of three formation cycles at 0.1C and after 100 cycles at 0.5C rate. The SEM images show no change in the particle's morphology after three cycles for both coated and uncoated electrodes. After 100 cycles, however, severe surface cracking was observed for the bare cathode particles, while no damage was recognized in the coated active mass. The extensive surface cracking supports the assumption that the degradation of the cathodes is caused by attacks of acidic species like HF at the electrode–electrolyte solution interface.^[^ ^25^ ^]^ The interactions between the acidic species in solution and LCO lead to dissolution of Co ions from the cathode surface and to capacity fading during prolonged cycling.^[^ ^25^, ^26^ ^]^ In contrast, the presence of the RbAlF_4_ coating seems to provide effective protection to the cathodes' active mass by preventing detrimental side reactions with the electrolyte solution.

Indeed, inductively coupled plasma‐mass spectroscopy (ICP‐MS) measurements of lithium anodes after 50 cycles reveal nearly 2.4 times larger amounts of cobalt traces on them when the cells contained bare cathodes, compared to similar experiments with coated cathodes. The F1s photo‐electron spectra (XPS) show distinguishable differences between the bare (Figure 5c) and coated (Figure 5g) electrodes. After three cycles, the spectra of both cathodes consist of a peak at 685 eV attributed to LiF and a second peak at 687.7 eV, which corresponds to the PVdF binder. Yet, the ratios between the two peaks reveal a higher relative intensity of the LiF in spectra of the bare LCO. Figure 5c shows a significant increase in this relative intensity after 100 cycles due to additional formation of LiF‐containing surface films. In general, the formation of LiF occurs via neutralization reactions between HF and hydroxyl groups on the LCO surface followed by production of H_2_O,^[^ ^3^ ^]^ or by disproportionation of LiCoO_2_, which forms Li_2_O that interacts with HF to give LiF and water.^[^ ^26^ ^]^ As both routes are associated with generation of H_2_O, they have an autocatalytic character: formation of LiF induces further production of water that forms HF. Without the protective coating, this process is expected to proceed and results in the formation of thick and resistive surface films that consist of LiF. As this reaction is followed by HF generation, further dissolution of Co from the cathode is likely to occur. In sharp contrast, a new peak at ≈686 eV attributed to Li*_x_* PO*_y_* F*_z_* was detected in the spectra of the coated cathodes after 100 cycles, while the intensity of the LiF peaks remains nearly unchanged. The favorable formation of Li*_x_* PO*_y_* F*_z_* over LiF results in the development of a stable passivation layer that hinders parasitic interactions with the electrolyte solution and does not facilitate detrimental H_2_O formation. As demonstrated in the Co 2p spectra in Figure S6, Supporting Information, the uncoated sample exhibited a higher Co^2+^ peak intensity (located at 783 eV) than Co^3+^ (783 eV). As suggested by previous studies,^[^ ^27^, ^28^ ^]^ this peak can be attributed to the formation of CoF_2_ induced by HF attacks on LCO surface. In contrast, smaller amount of the Co^2+^ (relative to Co^3+^) was detected for the coated LCO which demonstrated effective protection for undesirable parasitic reactions.

The improved surface properties of the coated samples are well reflected by impedance spectroscopic measurements (EIS) presented in Figure S7, Supporting Information, as Nyquist plots: before cycling slightly larger impedance values were detected for the coated cathodes compared to the bare ones which can be ascribed to the presence of the RAF coating on the LCO surface (see Figure S7a, Supporting Information). After 3 formation cycles (in 0.1C) similar impedance values were recorded for both samples (Figure S7b, Supporting Information). In contrast, after additional 50 cycles (in 1C) a substantial decrease in the charge transfer resistance (*R*_ct_) was measured for the coated cathodes, while an additional increase of the *R*_ct_ was recognized for the bare LCO cathodes (Figure S7c, Supporting Information). The lower impedance of the former (coated) electrodes is attributed to the stabilization of the SEI layer upon cycling by formation of more conductive surface spices or by compactization and homogenization of the surface films thus formed as was indicated by various parallel studies.^[^ ^29^, ^30^, ^31^ ^]^

The (003) XRD reflections of the bare and Rb—Al—F coated samples (after 3 and 100 cycles) are presented in Figures 5d and 5h, respectively (full XRD patterns and (015) peak for both samples are shown in Figure S8, Supporting Information). A positive shift from ≈19.2° to ≈19.5° was identified for the uncoated sample, while no significant changes in the peak location were detected for the coated cathode. In general, during charging to high voltage (beyond 4.5 V) the LCO cathodes undergo phase transitions from O3 (octahedral triple‐phase) to H1‐3 (hybrid phase of octahedral O1 and O3 phases).^[^ ^32^ ^]^ These phase transitions are accompanied by dissolution of the oxidized Co atoms and their possible redox reactions with solution species, which results in irreversible structural changes and loss of active Li insertion sites. As shown in Figure 5d, after 100 cycles the structure of the bare cathode was not fully recovered and remained partially oxidized. In contrast, the coated samples displayed fully reversible changes in the XRD patterns implying a high structural stability of the coated cathodes.

This finding is well correlated with our ICP and SEM observations in which significant Co ions dissolution followed by particles pulverization was identified. Cross sections analysis of bare and coated LCO particles taken from cycled electrodes (cut by focused ion beam ‐ FIB) revealed pronounced cracking of the particles' interior for the uncoated active mass, while much less cracks were detected for particles that belonged to coated LCO electrodes (see Figure S9, Supporting Information)

Based on the above results, it is clear that the combination of Rb‐based coating and electrolyte solutions containing fluorinated cosolvents provides enhanced stability to high voltage LCO cathodes. Yet, most previous studies focus on the performance of the 4.6 V cathodes in a half‐cell configuration using Li metal as the counter electrode. Using Li metal anodes enables indeed to concentrate on the cathodes' behavior, at least when the cycle life test is not too long. Since lithium anodes are unlimited sources for active Li‐ions in the cells, one of the major effects of side reactions, namely, depleting the amount of active Li ions in the cells, cannot be measured in the so‐called half cells testing. Nevertheless, application of high charging potentials on cells containing Li metal anodes is very problematic for them as well. High charging voltage results in oxidation of the electrolyte solution, irreversible consumption of solutions species in side reactions, and migration of oxidation products to the Li anode side. This “cross‐talk” between the electrodes leads to thickening of the surface films on the Li anode (via reduction of solution oxidation products on the Li surface). This leads to development of resistive films on the Li anodes, worsening the free transport of Li ions in the cells, and consequently their observed capacity fading.^[^ ^33^ ^]^

**Figure 6:**
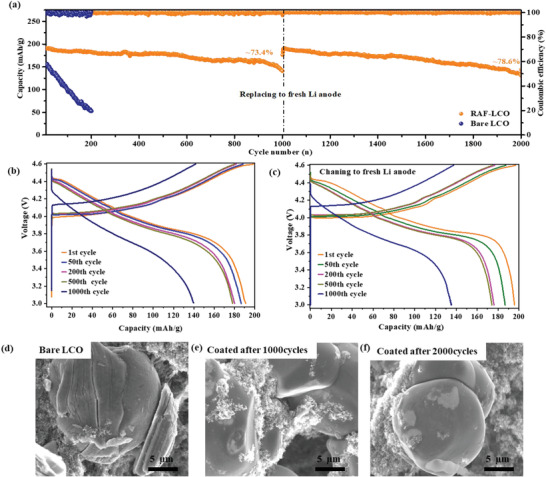
Influence of the Li anodes on cells' performance: a) long‐term cycling of bare and coated LCO cathodes performed at 1C rate. The GCD profiles recorded during 1000 cycles before and after Li anodes' replacement are shown in (b) and (c), respectively. SEM images are shown for the bare LCO after 200 cycles (d) and for the coated LCO after 1000 cycles (e) and additional 1000 cycles with fresh Li foil anodes after anodes' replacement (f).

Hence, the status of the cathodes affects the performance of the anodes in the cells. We examined the effect of the LCO coatings on the Li anodes. The following experiments were performed: electrodes comprising coated and uncoated LCO were cycled at 1C in Li‐LCO cells. When sharp capacity fading was observed, the Li anodes were replaced by fresh Li foils, while keeping the originally cycled cathodes in the cells. As shown in **Figure** **6**a, an initial capacity of 190 mAh g^−1^ was obtained with the coated cathodes. After 1000 cycles, a 73.4% capacity retention (143 mAh g^−1^), followed by accelerated capacity loss (as indicated by the changes in the capacity slope) was observed. At this stage, the cells were opened and reassembled with fresh Li anodes. As can be seen, after a gradual increase in capacity, which may be ascribed to electrolyte solution impregnation in the composite cathodes a fully restored capacity (190 mAh g^−1^) was observed, while after additional 1000 cycles, slightly lower capacity retention of 70.0% was recorded. In sharp contrast, fast capacity fading was observed for cells comprising the bare cathodes, with only 33% capacity retention. In this case, even after changing to fresh Li anodes, the capacity of the cells could not be even partially restored. Examination of the GCD voltage profiles of cells with coated cathodes, recorded before and after Li anodes replacement (Figure 6b and 6c, respectively) reflects the capacity fading and increased voltage hysteresis after 1000 cycles. By changing to fresh Li foil anodes, the voltage profiles of the cycled cells were fully restored in terms of both high capacity and low hysteresis.

These measurements confirm that a major failure mechanism for cells with the coated LCO cathodes is related to the Li‐anodes rather than to the cathodes' stability. Indeed, no changes in the morphology of the coated LCO particles were observed after 1000 and even 2000 cycles as presented in Figure 6e,f, while severe particle cracking can be readily recognized for the uncoated LCO. These findings may offer a different viewpoint regarding the examination of degradation phenomena of cells based on high voltage cathodes. The “cross‐talk” between the cathode and the anode in the cells may play a significant role in the failure mechanism of the Li‐anode. This degradation route involves dissolution of Co cations from the cathode's active mass, it is accelerated at high voltage (oxidation processes form acidic species like HF that attack the LCO, leading to protons, i.e., Co ions exchange). The Co cations migrate to the anode and their reduction processes there worsen the Li anode's passivation, which leads to enhanced side reactions and the development of high impedance in the cells. The application of high potentials leads to strains and stresses in the LCO active mass, that form cracks. Cracks expose fresh LCO surface to solution species, leading to enhanced side reactions, an autocatalytic scenario. Appropriate protective coating, as suggested herein, well avoids all the above‐described detrimental processes. We conclude that any study of high voltage Li cells, should include a thorough investigation of the anode side as well, and of the way side reactions at the cathode side, affect detrimentally the Li anode's performance. What we show herein with Li metal anodes should be much more severe with full cells containing graphite anodes, because increased side reactions should consume rapidly the active Li ions in these cells which are limited by the cathode's active mass, thus adding more options for failure. Indeed, substantially faster capacity fading was observed for full cells comprising LCO cathodes and graphite anodes. Yet significantly better capacity retention (≈90%) was observed for the graphite coated LCO cells after 100 cycles, whereas the bare (uncoated) LCO‐based cells exhibited much faster capacity fading (retention ≈61% only, after 100 cycles, see Figure S10, Supporting Information).

## 3 Conclusions

The current work presents a new coating based on Rb—Al—F (RAF) that enables improved stability of LCO cathodes in DFEC‐based electrolyte solutions at a high charging voltage of 4.6 V at both room and elevated temperatures. The enhanced stability of the LCO cathode is attributed to the protective nature of the RbAlF4 coating, which prevents HF attack on the LCO surface and mitigates Co dissolution and surface cracking. In this regard, the effect of additional metal aluminum fluorides (for example Na—Al—F, K—Al—F, etc.) should be carefully evaluated by theoretical and experimental studies (in a follow‐up work). We demonstrated that for cells with coated cathodes, the limiting factor in the cells' stability is the Li anodes, which undergo massive passivation by inevitable electrolyte solution reduction upon long‐term cycling. The coated cathodes in Li cells remain stable for 2000 cycles. Further development of high voltage LCO electrodes, therefore, requires not only the application of an effective coating on the cathode side and the use of suitable electrolyte solutions. Special efforts should be invested in developing stable and robust anodes' passivation.

## Conflict of Interest

The authors declare no conflict of interest.

## Supporting information

Supporting Information

## Data Availability

The data that support the findings of this study are available from the corresponding author upon reasonable request.
