# Supplementary material for: Highly Stable 4.6 V LiCoO2 Cathodes for Rechargeable Li Batteries by Rubidium‐Based Surface Modifications
Source: Adv Sci (Weinh). 2022 Oct 17;9(33):2202627. doi: 10.1002/advs.202202627 (PMC9685477; doi:10.1002/advs.202202627)
Supplement: Supplementary file 1 — Supporting Information [file ADVS-9-2202627-s001.pdf]

ADVANCED SCIENCE
Open Access

Supporting Information

for *Adv. Sci.*, DOI 10.1002/advs.202202627

Highly Stable 4.6 V $LiCoO_2$ Cathodes for Rechargeable Li Batteries by Rubidium-Based Surface Modifications

Tianju Fan, Yujie Wang, Villa Krishna Harika, Amey Nimkar, Kai Wang, Xiaolang Liu, Meng Wang, Leimin Xu, Yuval Elias, Hadar Sclar, Munseok S. Chae, Yonggang Min, Yuhao Lu*, Netanel Shpigel* and Doron Aurbach**

Supporting Information

Highly stable 4.6V $LiCoO_2$ cathodes for rechargeable Li batteries by rubidium-based surface modifications

Tianju Fan[1,2], Yujie Wang[2], Villa Krishna Harika[1], Amey Nimkar[1], Kai Wang[3], Xiaolang Liu[3], Meng Wang[3], Leimin Xu[3], Yuval Elias[1], Hadar Scalar[1], Munseok S. Chae[1], Yonggang Min[2], Yuhao Lu[3]*, Netanel Shpigel[1]* and Doron Aurbach[1]

[1]Department of Chemistry, Bar-Ilan University, Ramat-Gan 5290002, Israel.

[2]School of Materials and Energy, Guangdong University of technology, Guangzhou, Guangdong 510006, China.

[3]Ningde Amperex Technology Limited, Ningde, Fujian 352100, China.

*nshpigel@gmail.com, Luyuh@ATLBattery.com

Experimental Section

Materials

The $Al(NO_3)_3•9H_2O$ (99.99%, Aladdin), $RbSO_4$ (99.99%, Aladdin), NH_4F (99.99%, Aladdin), and $LiCoO_2$ (99.5%, tap density of 2.9g/cm^3 ,ATL Corporation) were used without purification.

Syntheses of RAF/AF/RF coated $LiCoO_2$ particles:

Calculated amounts of $Al(NO_3)_3•9H_2O$, $RbSO_4$, and NH_4F were dissolved in 35 mL of deionized water and put under stirring for 2h (for example, in the case of forming 2% RAF-LCO, ($Al(NO_3)_3•9H_2O$: $RbSO_4$: NH_4F = 28.37 mg:20.19 mg:28.02mg was used). 1 g of $LiCoO_2$ was added to the solution and left for overnight stirring. The as-prepared mixture was transferred to a Teflon-lined autoclave, and a hydrothermal reaction was conducted at 160°C for 5 h. The obtained product was washed with deionized water and overnight dried at 80°C. Finally, the powder was collected and calcined at 700°C for 5 h in argon gas with a heating rate of 5°C min^{-1} from room temperature.

Electrodes Preparation and cell assembly:

Slurries consist of 80% active material particles (coated or Bare LCO), 10 wt% super P and 10 wt% polyvinylidene fluoride (PVDF, 99.5%, Arkema) dissolved in an appropriate amount of N-methyl-1,2-pyrrolidone (NMP MTI corporation KJ GROUP) were spread on Al foils. The thickness of the coatings

was adjusted to 100 - 150 µm. After overnight drying at 120°C in a vacuum oven, the electrode was punched and assembled into (CR2032) coin cells, where lithium foils were used as counter electrodes and polypropylene (PP, Celgard 2400) separator, soaked by 90 µL of the selected electrolyte solutions. The loading of the active mass in the cathodes was set to be ~ 6 mg cm^{-2}. The electrolytes used in this study are ethylene carbonate/ethyl-methyl carbonate (EC/EMC, 3:7 by volume – denoted as LP57), and 1M.

Instrumentation

Galvanostatic cycling was performed using Arbin battery cyclers. ***High-resolution SEM imaging*** was performed using a Magellan XHR 400L FE-SEM (FEI Company) equipped with an EDS detector (Oxford Instruments). ***XRD measurements*** were performed using an AXS D8 ADVANCE diffractometer (Bruker Inc., Germany) using Cu Kα radiation, a Ni filter, and a scintillation counter. ***Inductive coupled plasma (ICP)*** **analysis** was performed using a Spectro ARCOS ICP-OES Multiview FHX22 instrument. ***High-resolution TEM imaging*** was conducted by a JEOL-JEM 2100 electron microscope with LaB6 emitter operating at 200 kV equipped with a Thermo Scientific Ultra Dry EDS detector

Computational methods：

Density functional theory (DFT) analyses were performed by Vienna Ab-initio Simulation Package (VASP) package[1–3]. The generalized gradient approximation (GGA) exchange-correlation functional, parameterized by Perdew, Burke, and Enzerof (PBE)[4] was applied with projected augmented wave (PAW) method[56] The valence configurations of each elements were Co-$3d^8 4s^1$, O-$2s^2 2p^4$, Li-1 $s^2 2s^1$, F-$2S^2 2p^5$, Al-$3s^2 3p^1$ and Rb-$4s^2 4p^6 5s^1$. Plane-wave basis sets were used with a cut-off energy of 500 eV, and a Monkhorst-Pack[7] kpoint mesh of 2 × 2 × 1 was adopted. Higher cut-off energy and denser k-mesh were tested, and the energy differences were less than 5meV/atom. An effective U parameter of 3.32 eV [8] was adopted to deal with the repulsive Coulomb interactions between Co d orbitals. The cluster expansion and Special Quasirandom Structures (SQS) methods as implemented in the ATAT [9] package was used to generate the Rb-Al-F-LCO solid solution structure. The convergence tolerances of the SCF calculations were set to be 10^{-5} eV for the total energy. The structure relaxations were performed until all forces on each atom were less than 0.02 eV/Å, and the total stress tensor was within 0.1 GPa. Long-range vdW interactions are accounted for using the Grimme DFT-D3 scheme[10]. Spin polarization was considered in all calculations. To simulate the pure LCO configuration, a 3 × 3 × 1 $LiCoO_2$ supercell was used (see fig. 1a). There were 108 atoms ($Li_{27}Co_{27}O_{54}$) in the pure LCO supercell. To simulate the LCO-Rb-Al-F solid solution, a supercell with 90 atoms ($Li_6Rb_3Co_{18}Al_9O_{36}F_{18}$ with a formula $Li_{2/9}Rb_{1/9}Co_{2/3}Al_{1/3}O_{4/3}F_{2/3}$) was applied (see fig.1b). The concentration 1/3 of Al was determined by Qian et al [8] who calculated the

formation energies of $LiAl_xCo_{1-x}O_2$ with different x, and found that $LiAl_{1/3}Co_{2/3}O_2$ showed the lowest energy. The formation energy of the pure $LiCoO_2$ was defined as

$$E_{form\_LCO} = E_{LCO} - E_{CoO2} - E_{Li}$$

and the formation energy of LCO-Rb-Al-F solid solution was defined as:

$$E_{form\_LCO\text{-}Rb\text{-}Al\text{-}F} = E_{LCO\text{-}Rb\text{-}Al\text{-}F} - 2/9E_{Li} - 1/9E_{Rb} - 2/3E_{CoO2} - 1/3E_{Al} - 1/3E_{F2}$$

where E_{LCO} and $E_{LCO\text{-}Rb\text{-}Al\text{-}F}$ were bulk energies of $LiCoO_2$ and $Li_{2/9}Rb_{1/9}Co_{2/3}Al_{1/3}O_{4/3}F_{2/3}$, E_{Li}, E_{Rb}, and E_{Al} were energies of elemental Li, Rb and Al, and E_{F2} was the energy of F_2 molecule.

The surface energies of LCO and LCO-Rb-Al-F solid solution were defined as:

$$E_{surf} = (E_{slab} - E_{bulk}) / 2A$$

where E_{slab} was the energy of the slab with surfaces, E_{bulk} was the energy of the bulk system, and A was the surface area.

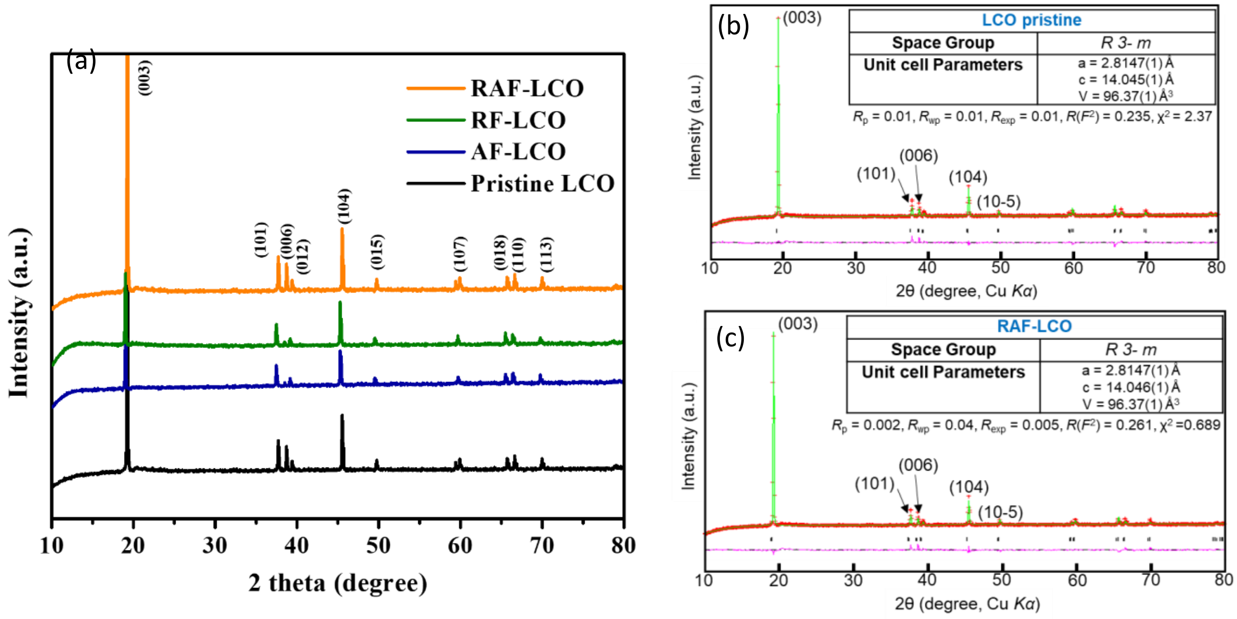

Fig S1. **(a)** XRD patterns of RAF-LCO, AF-LCO, RF-LCO, and pristine LCO **(b)** Rietveld Analysis of bare and RAF coated LCO.

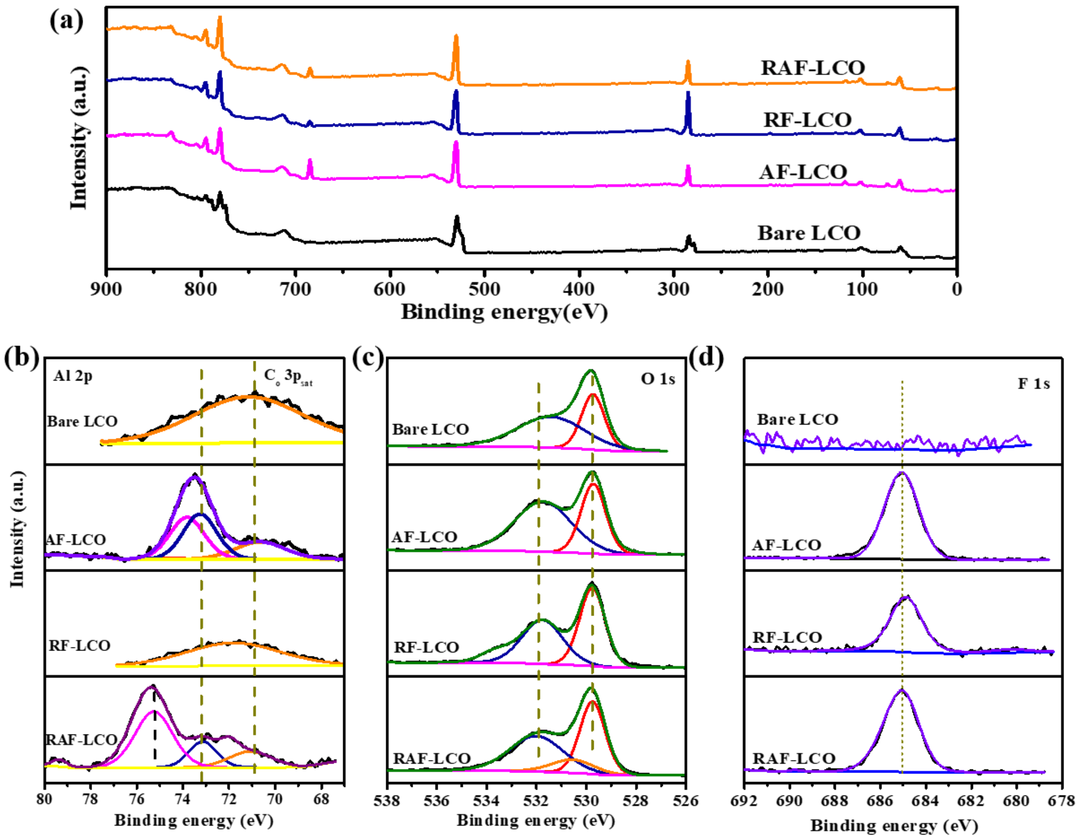

Fig.S2. Characterization of XPS data of Bare LCO and RAF-LCO electrodes (a). XPS scans of Al 2p peaks (including Co_{3psat} peak), the O 1s peaks of Bare LCO, AF-LCO, RF-LCO and RAF-LCO(b), and the O 1s peaks of bare LCO, AF-LCO, RF-LCO and RAF-LCO (c) the F 1s peak of bare LCO, AF-LCO, RF-LCO and RAF-LCO (d).

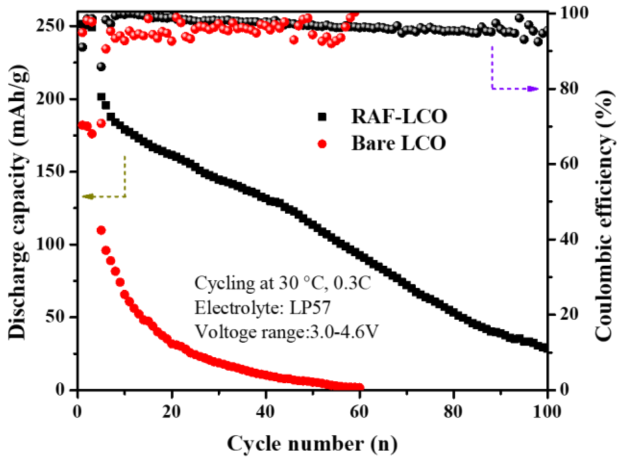

Fig.S3. Cycling performance of lithium cells with bare LCO or RAF-LCO electrodes with LP57 electrolyte solution at 30 °C in the voltage range of 3.0-4.6 V (vs. Li^+/Li) at the current density of 110 mA/g.

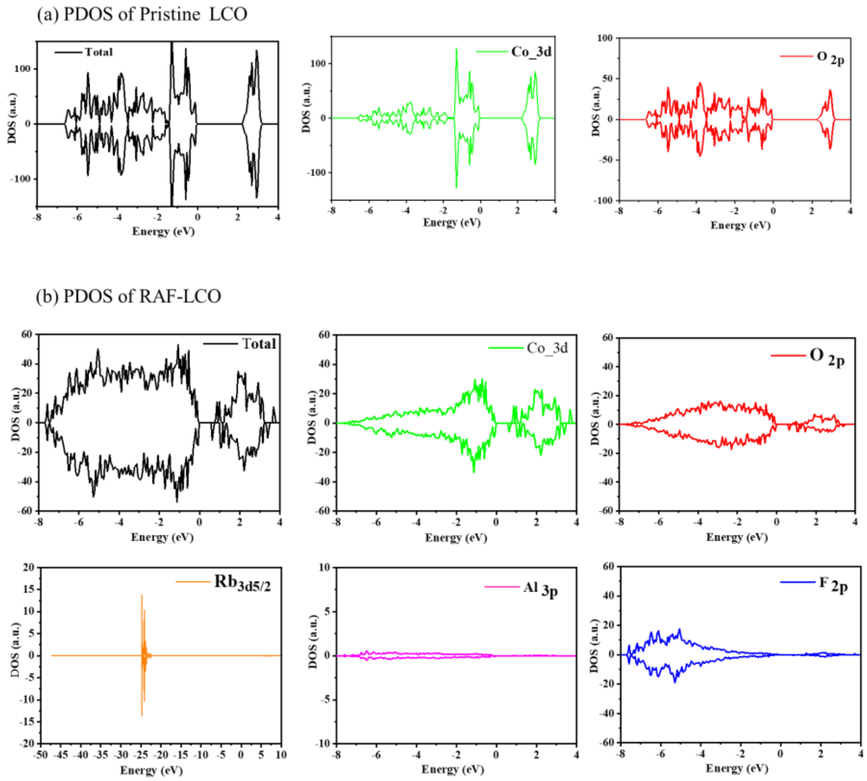

Fig.S4. Projected density of electronic states (PDOS) of (a) bare LCO (b) RAF-coated LCO.

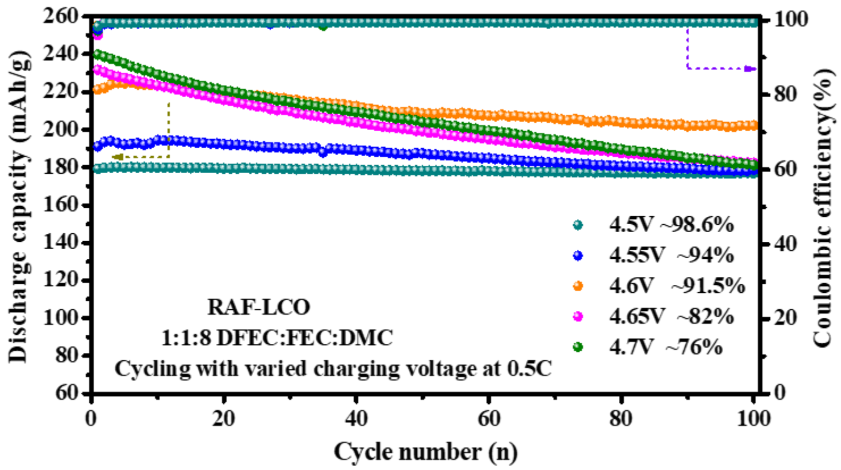

Fig.S5. A comparison of the cycling performance of Li cells containing RAF-LCO cathodes, as a function of the maximal charging potentials: 4.5V, 4.55V,4.6V,4.65V to 4.7V at 0.5C rates, using electrolyte solution containing 1M $LiPF_6$ 1:1:8 DFEC/FEC/DMC.

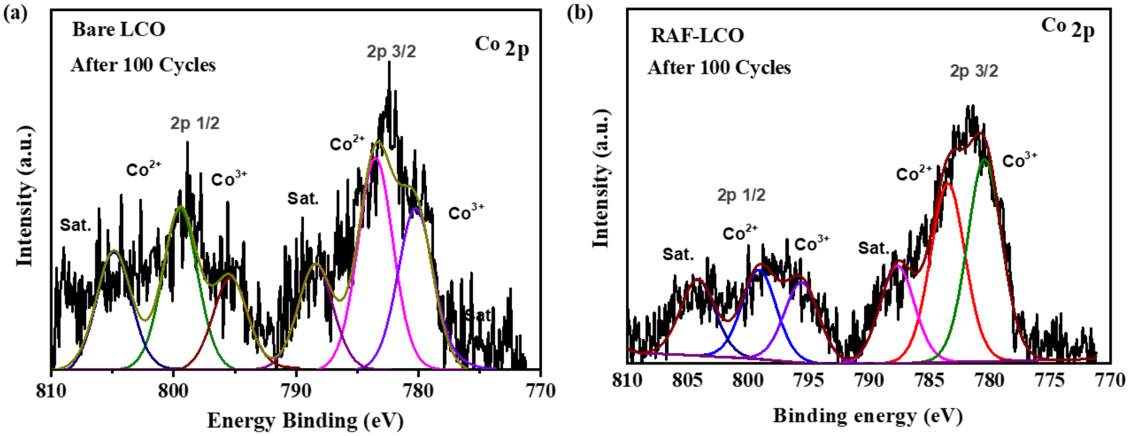

Fig.S6 Co 2P spectra collected for the bare **(a)** and the RAF coated electrodes **(b)** after 100 cycles

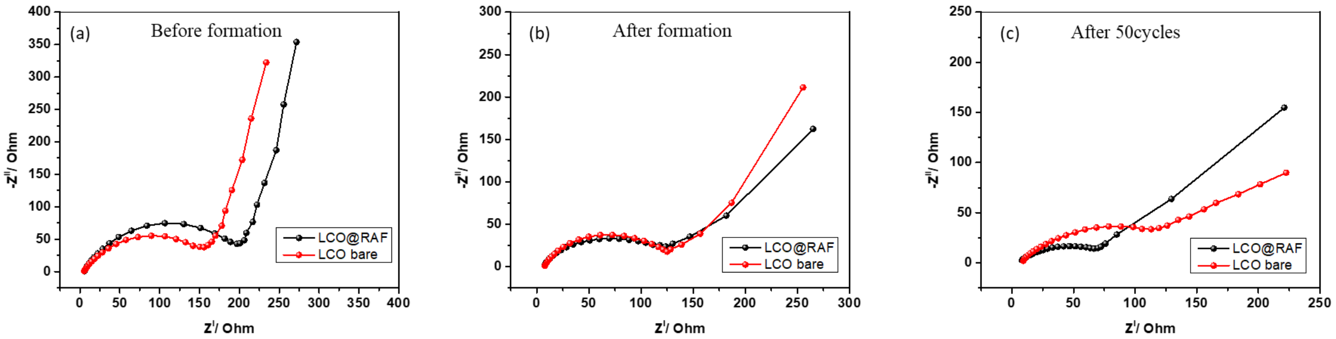

Fig.S7 Electrochemical impedance spectra (EIS) measured for the bare (red lines) and the coated (black lines) LCO electrodes at a fully lithiated state (a) before cycling (b) after 3 formation cycles at 0.1C (c) after 50 cycles at 1C

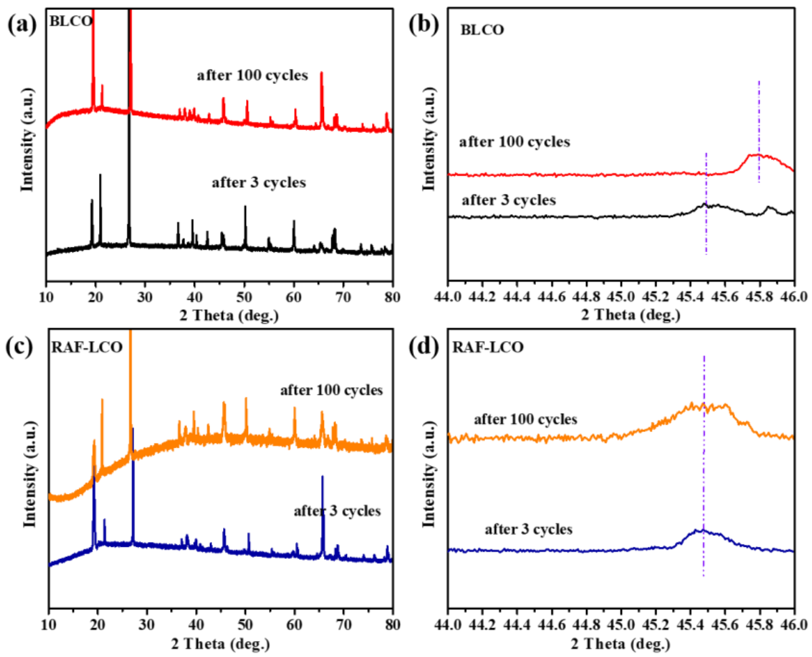

Fig.S8. The full XRD patterns and the (015) peaks of bare LCO (a) and 2% RAF coated LCO electrodes (c) after 3 cycles and after 100 cycles at a current density 22 mA/g and after 100 cycles at 73.0 mA/g.

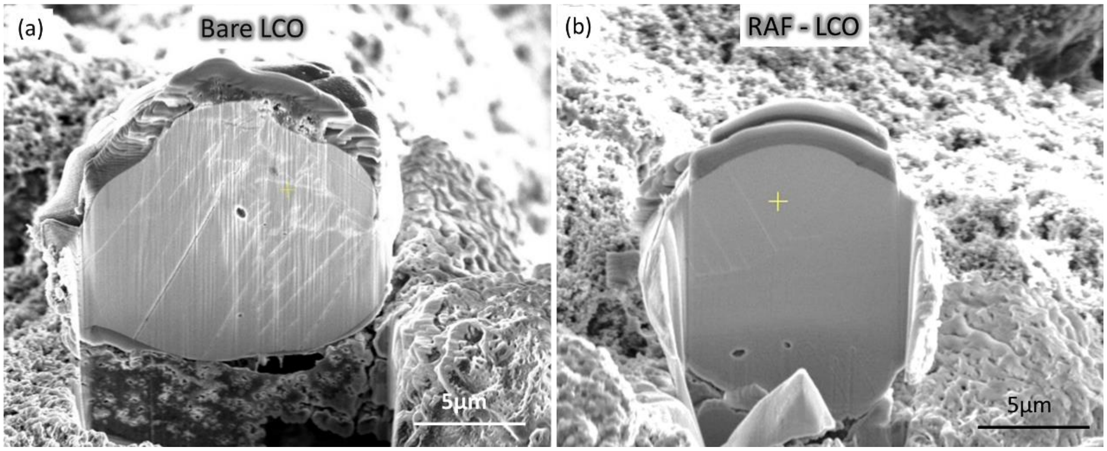

Figure S9 Cross sections analyses of **(a)** uncoated LCO and **(b)** RAF coated LCO after 100 cycles by SEM. The paricles were cut by a FIB technique.

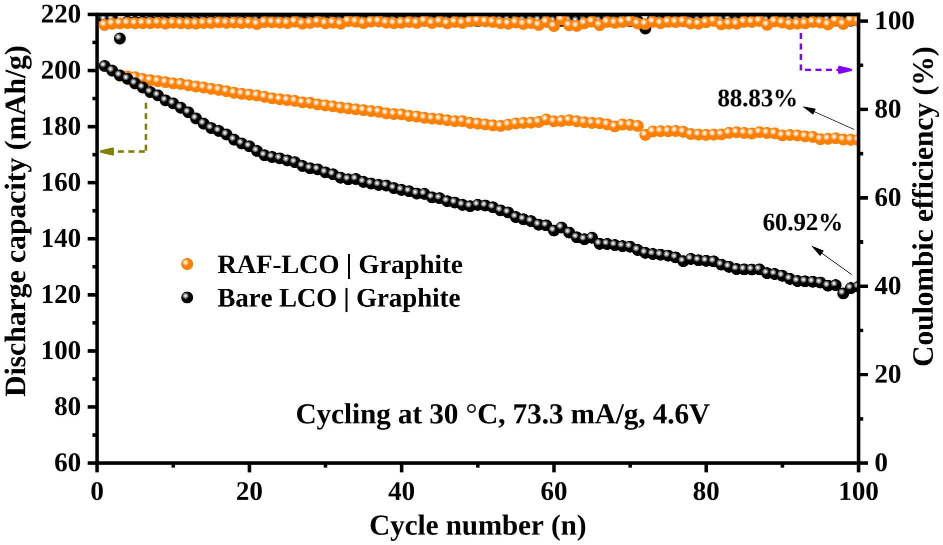

Figure S10 Cycling performance of full LCO graphite cells comprising bare or RAF coated cathodes' particles.

Table. S1 Comparison of electrochemical performance of RAF-LCO cathodes described in the state-of-the-art reported with high cut-off voltage-up to 4.6V.

No.	LCO with treatment	Electrolyte	Initial capacity mAh/g (rate)	Cycling performance (retention)	Reference
1	LATP-LCO	$LiPF_6$ EC/DEC 1:1	200 (0.5C)	100 (88%)	[11]
2	TMA-LCO	$LiPF_6$ EC/DEC 1:1	174 (0.5C)	100 (86%)	[12]
3	MgF_2 doped LCO $LM_{0.01}COF_{0.02}$	$LiPF_6$ EC/DMC 1:1	198 (1C)	100 (92%)	[13]
4	Se-LCO	1.2M $LiPF_6$ EC/DEC 1:1+ 2 wt% vinylene carbonate	218 (70 mA/g)	500 (80.8%) 120 (86.7%)	[14]
5	Li-Al-P coated on LCO	$LiPF_6$ EC/DMC 1:1	200 (0.7C)	200 (79.2%)	[15]
6	T-LCO	sulfonamide-based electrolyte 1 m LiFSI/DMCF3SA	220 (0.2 C)	100 (85%)	[16]
7	Mg-LCO	1M $LiPF_6$ EC/DEC +0.5% KSeCN	193.2 (1C)	1000 (70.4%)	*[17]*
**8**	**RAF-LCO**	**$LiPF_6$ DFEC/FEC/DEC 1:1:8**	**221.18 (0.3C)** **218.03 (0.5C)** **198.5 (1C)**	**100 (92.8%)** **500 (~81%)** **2000 (~76%)**	***This work***

References

(1) Kresse, G.; Hafner, J. *Ab Initio* Molecular Dynamics for Liquid Metals. *Phys. Rev. B* **1993**, *47* (1), 558. https://doi.org/10.1103/PhysRevB.47.558.

(2) Kresse, G.; Hafner, J. *Ab Initio* Molecular-Dynamics Simulation of the Liquid-Metal–Amorphous-Semiconductor Transition in Germanium. *Phys. Rev. B* **1994**, *49* (20), 14251. https://doi.org/10.1103/PhysRevB.49.14251.

(3) Kresse, G.; Furthmüller, J. Efficiency of Ab-Initio Total Energy Calculations for Metals and Semiconductors Using a Plane-Wave Basis Set. *Comput. Mater. Sci.* **1996**, *6* (1), 15–50. https://doi.org/10.1016/0927-0256(96)00008-0.

(4) Perdew, J. P.; Burke, K.; Ernzerhof, M. Generalized Gradient Approximation Made Simple. *Phys. Rev. Lett.* **1996**, *77* (18), 3865. https://doi.org/10.1103/PhysRevLett.77.3865.

(5) Kresse, G.; Joubert, D. From Ultrasoft Pseudopotentials to the Projector Augmented-Wave Method. *Phys. Rev. B* **1999**, *59* (3), 1758. https://doi.org/10.1103/PhysRevB.59.1758.

(6) Blöchl, P. E. Projector Augmented-Wave Method. *Phys. Rev. B* **1994**, *50* (24), 17953. https://doi.org/10.1103/PhysRevB.50.17953.

(7) Monkhorst, H. J.; Pack, J. D. Special Points for Brillouin-Zone Integrations. *Phys. Rev. B* **1976**, *13* (12), 5188. https://doi.org/10.1103/PhysRevB.13.5188.

(8) Qian, J.; Liu, L.; Yang, J.; Li, S.; Wang, X.; Zhuang, H. L.; Lu, Y. Electrochemical Surface Passivation of LiCoO2 Particles at Ultrahigh Voltage and Its Applications in Lithium-Based Batteries. *Nat. Commun.* **2018**. https://doi.org/10.1038/s41467-018-07296-6.

(9) van de Walle, A. Multicomponent Multisublattice Alloys, Nonconfigurational Entropy and Other Additions to the Alloy Theoretic Automated Toolkit. *Calphad* **2009**, *33* (2), 266–278. https://doi.org/10.1016/J.CALPHAD.2008.12.005.

(10) Grimme, S.; Antony, J.; Ehrlich, S.; Krieg, H. A Consistent and Accurate Ab Initio Parametrization of Density Functional Dispersion Correction (DFT-D) for the 94 Elements H-Pu. *J. Chem. Phys.* **2010**, *132* (15), 154104. https://doi.org/10.1063/1.3382344.

(11) Wang, Y.; Zhang, Q.; Xue, Z. C.; Yang, L.; Wang, J.; Meng, F.; Li, Q.; Pan, H.; Zhang, J. N.;

Jiang, Z.; Yang, W.; Yu, X.; Gu, L.; Li, H. An In Situ Formed Surface Coating Layer Enabling $LiCoO_2$ with Stable 4.6 V High-Voltage Cycle Performances. *Adv. Energy Mater.* **2020**, *10* (28). https://doi.org/10.1002/AENM.202001413.

(12) Zhang, J. N.; Li, Q.; Ouyang, C.; Yu, X.; Ge, M.; Huang, X.; Hu, E.; Ma, C.; Li, S.; Xiao, R.; Yang, W.; Chu, Y.; Liu, Y.; Yu, H.; Yang, X. Q.; Huang, X.; Chen, L.; Li, H. Trace Doping of Multiple Elements Enables Stable Battery Cycling of $LiCoO_2$ at 4.6 V. *Nat. Energy 2019 47* **2019**, *4* (7), 594–603. https://doi.org/10.1038/s41560-019-0409-z.

(13) Kong, W.; Zhang, J.; Wong, D.; Yang, W.; Yang, J.; Schulz, C.; Liu, X. Tailoring Co3d and O2p Band Centers to Inhibit Oxygen Escape for Stable 4.6 V $LiCoO_2$ Cathodes. *Angew. Chemie Int. Ed.* **2021**, *60* (52), 27102–27112. https://doi.org/10.1002/ANIE.202112508.

(14) Zhu, Z.; Wang, H.; Li, Y.; Gao, R.; Xiao, X.; Yu, Q.; Wang, C.; Waluyo, I.; Ding, J.; Hunt, A.; Li, J. A Surface Se-Substituted $LiCo[O_{2-\delta}Se_{\delta}]$ Cathode with Ultrastable High-Voltage Cycling in Pouch Full-Cells. *Adv. Mater.* **2020**. https://doi.org/10.1002/adma.202005182.

(15) Wang, X.; Wu, Q.; Li, S.; Tong, Z.; Wang, D.; Zhuang, H. L.; Wang, X.; Lu, Y. Lithium-Aluminum-Phosphate Coating Enables Stable 4.6 V Cycling Performance of $LiCoO_2$ at Room Temperature and Beyond. *Energy Storage Mater.* **2021**, *37*, 67–76. https://doi.org/10.1016/J.ENSM.2021.01.031.

(16) Xue, W.; Gao, R.; Shi, Z.; Xiao, X.; Zhang, W.; Zhang, Y.; Zhu, Y. G.; Waluyo, I.; Li, Y.; Hill, M. R.; Zhu, Z.; Li, S.; Kuznetsov, O.; Zhang, Y.; Lee, W. K.; Hunt, A.; Harutyunyan, A.; Shao-Horn, Y.; Johnson, J. A.; Li, J. Stabilizing Electrode–Electrolyte Interfaces to Realize High-Voltage Li||$LiCoO_2$ Batteries by a Sulfonamide-Based Electrolyte. *Energy Environ. Sci.* **2021**, *14* (11), 6030–6040. https://doi.org/10.1039/D1EE01265G.

[17] Fu, A., Lin, J., Zhang, Z., Xu, C., Zou, Y., Liu, C., ... & Zheng, J. Synergistical Stabilization of Li Metal Anodes and $LiCoO_2$ Cathodes in High-Voltage Li‖ $LiCoO_2$ Batteries by Potassium Selenocyanate (KSeCN) Additive. ACS Energy Letters, 7, 2022,1364-1373.
